# Sociodemographic disparities in postnatal care coverage at comprehensive health centers in Hamedan City

**DOI:** 10.3389/fpubh.2024.1329787

**Published:** 2024-07-22

**Authors:** Azam Maleki, Farzaneh Soltani, Maryam Abasalizadeh, Rafat Bakht

**Affiliations:** ^1^Social Determinants of Health Research Center, Health and Metabolic Diseases Research Institute, Zanjan University of Medical Sciences, Zanjan, Iran; ^2^Department of Midwifery and Reproductive Health, Mother and Child Care Research Center, Hamadan University of Medical Sciences, Hamadan, Iran; ^3^Department of Midwifery and Reproductive Health, Student Research Committee, Hamadan University of Medical Sciences, Hamadan, Iran; ^4^Department of Midwifery and Reproductive Health, Hamadan University of Medical Sciences, Hamadan, Iran

**Keywords:** postnatal care coverage, midwifery services, women health, Iran, disparities - definition and paradigm

## Abstract

**Background:**

Postnatal care (PNC) is a crucial component of continuous healthcare and can be influenced by sociodemographic factors. This study aimed to examine the sociodemographic disparities in PNC coverage in Hamedan City.

**Methods:**

In this cross-sectional study, we utilized existing data recorded in the Health Integrated System of Hamedan City, located in Iran, from 2020 to 2021. The study population consisted of 853 women who were over 15 years old and had given birth within the past 42 days. The Health Equity Assessment Toolkit (HEAT) software was used to evaluate the socioeconomic inequalities in PNC coverage.

**Results:**

Overall, 531 (62.3%) of the women received three postnatal visits. The absolute concentration index (ACI) indicates that women aged 20–35 years, illiterate women, housewives, insured individuals, and urban residents experience a higher magnitude of inequality in PNC coverage. The negative values of the ACI suggest that the health index is concentrated among disadvantaged groups, with educational level inequalities being more pronounced than those related to age.

**Conclusion:**

Postnatal care coverage among mothers was relatively adequate; however, sociodemographic inequalities existed in the utilization of PNC services. It is recommended that policymakers make efforts to increase access to PNC services for mothers from low socio-economic groups.

## Introduction

Most maternal deaths occur during the postnatal period, and an estimated 2.8 million babies die within the first month of life (1). Postnatal care (PNC) services are crucial for improving outcomes for mothers and infants in low- and middle-income countries (2). Inadequate PNC can expose mothers to risks such as postpartum bleeding, eclampsia, puerperal infection, thromboembolic disease, breastfeeding problems, and psychological issues such as depression (3). In some countries, PNC coverage is relatively poorer compared to other maternal and child care services (4). The World Health Organization (WHO) recommends that mothers and their newborns receive postnatal care within 24 h of birth, on the third day, during the second week, and at 6 weeks postpartum (5).

In Iran, postpartum care is typically provided at governmental and nongovernmental facilities, with all services delivered outside the home. Governmental postnatal care services are provided free of charge and include the prevention, early detection, and treatment of complications and diseases, as well as counseling on breastfeeding, birth spacing, immunization, and maternal nutrition (6). Improving social conditions and facilitating access to health services and education can play a valuable role in ensuring the health of mothers and children, who are among the most vulnerable groups.

Considering the necessity of PNC as an essential strategy to save the lives of mothers and newborns, it is crucial to identify the factors that prevent mothers and infants from benefiting from postnatal care (6). Health inequality, defined as the disproportionate concentration of people with specific health behaviors in certain demographic subgroups, remains a significant challenge for health systems, especially in low-income countries (7). For instance, mothers with lower levels of education and income are much less likely to initiate and continue breastfeeding than those from higher socioeconomic classes (8).

Global research has identified various factors influencing the use of PNC services, with varied outcomes, including maternal age, education level, occupation, place and method of delivery, number of pregnancies, and awareness of PNC services (9, 10). Economic and social determinants can affect the receipt of these essential services or lead to adverse health consequences during the postnatal period. Individuals with lower socioeconomic status are less likely to receive routine healthcare or preventive health advice and have a higher incidence of adverse health outcomes (11, 12).

The results of the FiNaL Study showed that women belonging to low socioeconomic levels face significant deprivations in terms of access to formal and informal breastfeeding support and even access to information (13). A study conducted in Nigeria classified the determinants of PNC services into family and community levels, which include education level, financial status, urban or rural residence, religion, source of information, mother’s age, and previous experience with health services (14). Another study on determinants affecting maternal health outcomes in Ghana revealed that rural residents are less likely than urban residents to undergo PNC (15).

It is important to note that the variables affecting PNC service use differ based on socio-cultural factors within a particular community. These differences may be attributed to factors such as access to health facilities, intentional government interventions, geographic location, and cultural practices. Identifying sociodemographic factors associated with PNC utilization can help health planners design and implement evidence-based interventions to strengthen the health system and improve access to and use of PNC services. This study aimed to investigate the social and economic inequalities in postnatal care coverage among mothers in Hamedan City.

## Methods

### Study design and setting

In this cross-sectional study, existing data from the Health Integrated System of Hamedan City, located in Iran, for the years 2020–2021 were analyzed to assess socioeconomic inequalities in postnatal care (PNC) coverage. In Hamedan city, comprehensive health coverage is provided to all residents, both urban and rural, through health centers. All healthcare information, including PNC, is electronically recorded in the integrated health system. According to national guidelines, postpartum care includes three visits on days 1–3, 10–15, and 42–60 after delivery.

### Participants

The study population comprised women over 15 years old who had given birth within the past 42 days. A total of 853 women met these criteria and their data were included in the analysis.

### Sampling method

In Hamedan City, there are 17 comprehensive urban and 13 comprehensive rural health centers. For this study, a simple random sampling method was used to select 8 out of the 17 comprehensive urban health centers and 5 out of the 13 comprehensive rural health centers. Subsequently, all eligible participants within the selected centers were enrolled using the census sampling method.

### Data collection tools

#### Demographic and obstetrics checklist

The checklist included the following variables: age, occupation, education level, place of residence, insurance status, delivery method, experience of preterm delivery, and exclusive breastfeeding.

#### Postnatal coverage

The primary outcome of the present study is the number of postnatal visits within 42 days after delivery, which ranges from 0 to 3 visits according to national guidelines.

### Data analysis

Data analysis was performed using SPSS software version 16. Descriptive statistics were used to summarize the data. The relationship between qualitative variables was assessed using the Chi-square test, and a logistic regression model was employed to identify predictive variables associated with receiving postnatal care (PNC) at a 95% confidence level.

To assess inequalities in PNC coverage across socio-economic subgroups, the Health Equity Assessment Toolkit (HEAT) software version 4.0 (Beta) was utilized. Inequality was evaluated using the ACI (absolute concentration index) and R indices. The R ratio is a simple measure indicating relative inequality between two subgroups, with values greater than 1 indicating higher inequality (16). The ACI is a weighted measure that assesses inequality based on a natural ordering scale, where positive values indicate concentration among advantaged groups and negative values indicate concentration among disadvantaged groups. A higher absolute ACI indicates greater inequality (17).

## Results

### Demographic characteristics and their related factors

The results of data analysis indicated that the highest frequency distribution was observed among individuals aged 20–35 years (74.7%), unemployed individuals (91.3%), those who were illiterate (43.3%), urban residents (82.2%), and those with insurance coverage (95.3%).

Analysis of postnatal care (PNC) coverage based on demographic characteristics revealed that the highest frequency distribution of receiving at least one or more PNC visits was among individuals aged 20–35 years (74.4%), unemployed individuals (91.9%), those who were illiterate (44%), urban residents (82.1%), and those with insurance coverage (95.2%).

Furthermore, comparing PNC coverage according to employment status showed statistically significant differences (*p* = 0.014) (Table 1).

**Table 1:** Socio-economic characteristic of women in term of postnatal visits (*N* = 853).

Characteristics *N* (%)		Postnatal visits, *n* (%)		*p*-value
		0	1–3	
Age (y)				0.38
<20	67 (7.9)	1 (2.4)	66 (8.1)	
20–35	637 (74.7)	34 (81)	603 (74.4)	
>35	149 (17.5)	7 (16.7)	142 (17.5)	
Job				0.01^*^
No	779 (91.3)	34 (81%)	745 (91.9)	
Yes	74 (8.7)	8 (19%)	66 (8.1)	
Education				0.12
Illiterate/ Elementary	369 (43.3)	12 (28.6%)	357 (44)	
Secondary	244 (28.6)	14 (33.3%)	230 (28.4)	
Higher	240 (28.1)	16 (38.1%)	224 (27.6)	
Location Residence				0.84
Rural	152 (17.8)	7 (16.7%)	145 (17.9)	
Urban	701 (82.2)	35 (83.3%)	666 (82.1)	
Insurance				0.46
No	40 (4.7)	1 (2.4%)	39 (4.8)	
Yes	813 (95.3)	41 (97.6%)	772 (95.2)	

### Postnatal coverage and its related factors

More than 62% (531) of mothers received all three postnatal care visits. However, less than 5% (42) of women did not receive any postnatal care. The rate of exclusive breastfeeding up to 42 days after childbirth was 94.4% (805) (Table 2).

**Table 2:** The frequency of Postpartum Visits and Breast Milk Feeding (BMF) coverage.

Characteristics	*N* (%)
Postpartum visits	
0	42 (4.9)
1	101 (11.8)
2	179 (21.0)
3	531 (62.3)
Breast Milk Feeding (BMF)	
No	48 (5.6)
Yes	805 (94.4)

The prevalence of cesarean section was 53.2% (454), preterm labor was 7.6% (65), and unwanted pregnancy was 19.1% (690). Cesarean section was significantly associated with the number of postnatal care (PNC) visits, with a higher percentage of individuals not receiving any PNC at all following cesarean section compared to normal vaginal delivery (Table 3).

**Table 3:** The relationship between postnatal visits with obstetric characteristics of women (*N* = 853).

Characteristics *N* (%)		Postnatal visits, *n* (%)		*p-*value
		0	1–3	
Mode of delivery				<0.001^*^
CS	454 (53.2)	32 (7)	422 (93)	
NVD	399 (46.8)	10 (2.5)	389 (97.5)	
Intention to get pregnant				0.10
Unplanned	690 (80.9)	38 (5.5)	652 (94.5)	
Planned	163 (19.1)	4 (2.5)	159 (97.5)	
Preterm labor				0.18
No	788 (92.4)	41 (5.2)	747 (94.8)	
Yes	65 (7.6)	1 (1.5)	64 (98.5)	

CS, Cesarean section; NVD, Normal vaginal delivery.

A logistic regression model was employed to identify predictive variables associated with postnatal care (PNC). The results indicated that, after controlling for variables such as age, education, insurance, and residence of mothers, occupation showed a significant relationship with the frequency of PNC visits. Specifically, the odds of receiving care were 5.5 times higher among non-employed mothers compared to employed mothers (*p* = 0.018) (Table 4).

**Table 4:** The logistic regression model of the postnasal visits and demographics characteristics.

Backward model		B	S.E.	Wald	*p-*value^a^	Exp (B)	95% CI	
							Lower	Upper
Postnatal care coverage	Job (yes)	ref						
	Job (no)	0.977	0.413	5.583	0.01^*^	2.656	1.181	5.972

^a^Adjusted to age, education, location, and insurance variables.

### Socio-economic inequalities and PNC coverage

The results from Table 5 indicate that the coverage of at least one postnatal care visit was 95%. The absolute concentration index (ACI) highlights that the age group 20–35 years exhibits a higher magnitude of inequality in postnatal care (PNC) coverage compared to other age groups. Specifically, PNC coverage was more equitable in the age groups under 20 and over 35 years old.

**Table 5:** The percentage of affected population in terms of subgroups and inequality index.

Postnatal care coverage	Variable	Percentage of affected population	Risk Q1/Q5	Estimate of coverage	Ci 95% lower	Ci 95% upper	Aci	*R*	Setting average
	Age, y		0.45				−1.15	0.86	95
	<20	7.9		92.5	86	99			
	20–35	74.7		83.0	80.1	85.9			
	>35	17.5		79.8	73.3	86.3			
	Education		1.54				−3.87	0.80	
	Illiterate/elementary	43.3		89.7	86.5	92.8			
	Secondary	28.6		84.8	80.3	89.3			
	Higher	28.1		71.6	65.9	77.4			
	Insurance		–				–	1.01	
	No	4.7		82.5	70.1	94.8			
	Yes	95.3		83.2	80.7	85.8			
	Location residence		–				–	0.89	
	Rural	17.8		91.4	86.9	95.9			
	Urban	82.2		81.4	78.5	84.3			
	Job		–				–	0.75	
	No	91.3		85.1	82.6	87.6			
	Yes	8.7		63.5	52.2	74.7			

ACI, Absolute concentration index; R, Ratio®.

Regarding other demographic characteristics, inequalities in PNC coverage were more pronounced among the illiterate, housewives, insured individuals, and urban residents compared to other subgroups.

Furthermore, the R indices (R) revealed that the level of inequality was greater for the “insurance” variable (R > 1) and lower for variables such as age, education, occupation, and place of residence (R < 1). This suggests that inequality in PNC coverage is more pronounced based on insurance status compared to other demographic factors.

Negative values of the ACI indicate that the concentration of the health index is among disadvantaged populations, with educational inequalities being more prominent than age-related disparities (Table 5).

## Discussion

The results of the present study revealed that postnatal care (PNC) coverage in Hamedan, a city in Iran, was moderately high, with more than 62% of postpartum mothers receiving PNC, aligning with World Health Organization recommendations. Evidence suggests that widespread PNC availability could prevent between 10 and 27% of infant deaths (18). In this study, 37.7% of mothers received care 1–2 times, while approximately 5% did not receive any PNC at all. Limited PNC coverage is evident, particularly among economically disadvantaged families. For instance, in the Democratic Republic of the Congo, only 35% of women receive PNC (19), while in Nepal, PNC utilization stands at around 22% (20). In contrast, a study in Sri Lanka reported 76.9% coverage of recommended PNC visits (21).

In Iran, studies on PNC coverage are scarce, with data typically managed by the Ministry of Health. However, a longitudinal study in Tehran found high rates of prenatal care utilization (95%) and recommended visits (99%) among pregnant women (22).

Notably, a significant proportion of deliveries in the present study were by cesarean section (53.2%), and these mothers were less likely to receive PNC compared to those with vaginal deliveries. Similar findings on socio-economic inequality in PNC utilization after cesarean sections were reported in other studies (23), although some research in Ethiopia suggests cesarean delivery may increase PNC utilization due to perceived higher risks (24).

Iran has one of the highest cesarean section rates globally (25). Most cesarean sections in this study were likely emergency procedures, underscoring the critical need for post-cesarean care and follow-up due to higher complication risks compared to vaginal deliveries (26). Financial and cultural barriers may hinder cesarean-section mothers from accessing PNC, as seen in studies from Pakistan highlighting transportation and healthcare costs as deterrents (27).

Regarding demographics, PNC coverage was better among women under 20 and over 35 years old compared to those aged 20–35 years. In Iran, younger mothers (<20 years) receive heightened attention due to perceived higher pregnancy risks, while older mothers (>35 years) benefit from greater healthcare awareness and support. Similar findings on increased PNC utilization with advancing maternal age have been reported elsewhere (28, 29).

Education also significantly influenced PNC coverage, with lower rates among illiterate or minimally educated women compared to others (24–29). Education enhances health awareness and promotes health-seeking behavior, influencing service utilization (30).

Non-employed mothers in the study were 5.5 times more likely to receive PNC compared to employed counterparts, contrary to some expectations about economic independence and service access (29, 31). Rural residents also showed higher PNC coverage than urban counterparts, differing from findings in some studies (32, 33), but consistent with others (20, 34). Urban areas may offer better access to private healthcare facilities and health promotion programs, influencing PNC utilization (21).

Interestingly, while 95.3% of mothers in this study had health insurance, uninsured individuals had better PNC coverage in comprehensive health centers. The availability of health insurance allows mothers to choose private facilities or gynecologists’ offices, potentially reducing visits to public health centers. Efforts to improve PNC service quality in public centers could enhance overall access and utilization, regardless of socio-economic status.

### Strengths of study

The study benefits significantly from using data from the Health Integrated System, which likely offers a large sample size. This enables a comprehensive analysis of postnatal care (PNC) utilization and inequalities. The data within this system are objective and reliable, systematically collected as part of the healthcare system. This enhances the credibility of the study findings and reduces biases associated with self-reported data or survey responses. As a result, the study provides more accurate and representative insights into disparities in postnatal care. These findings can have profound implications for healthcare policies and interventions aimed at reducing inequality in PNC, thereby contributing to improved maternal and child health outcomes.

### Limitations of study

This study has several limitations that should be considered. Firstly, it is unable to examine disparities in postnatal care (PNC) provided at private facilities, focusing solely on public health centers. Secondly, as a cross-sectional analysis, it can establish associations between variables but cannot determine causation. Additionally, using secondary data introduces limitations such as potential exclusion of important factors like family support, proximity to healthcare facilities, and availability of healthcare providers. Maternal factors like residence patterns can also impact PNC access; for example, many Iranian mothers, particularly first-time mothers, often return to their parents’ homes postpartum, seeking care from family members, especially their mothers.

Furthermore, the study’s scope is limited to Hamedan City, a specific geographic area in Iran, which may restrict the generalizability of findings to other regions. To gain a comprehensive understanding of maternal and child health disparities, ongoing monitoring and evaluation of health indicators and related inequities are crucial. Future research that considers geographical and socio-economic factors could provide valuable insights into variations in postnatal care across diverse social and cultural contexts.

## Conclusion

Our analysis revealed significant disparities in postnatal care (PNC) utilization, particularly among specific demographic groups such as women aged 20–35, those with low education levels, housewives, insured individuals, and urban residents. By elucidating the intricate relationship between socioeconomic status and PNC coverage, our study offers valuable insights into global health inequalities. The negative values of the absolute concentration index (ACI) underscore the concentration of inadequate PNC coverage among marginalized individuals, emphasizing the urgent need for targeted interventions at a systemic level.

As we strive for universal health coverage and work toward achieving the Sustainable Development Goals, the findings of this research hold relevance Hamedan City, extending to regions worldwide grappling with similar challenges in ensuring equitable maternal healthcare. Addressing socioeconomic disparities in PNC coverage demands collective responsibility and coordinated action from policymakers, healthcare providers, and communities globally.

## Data availability statement

The raw data supporting the conclusions of this article will be made available by the authors, without undue reservation.

## Ethics statement

The studies involving humans were approved by the ethics committee of Hamadan University of Medical Sciences approved this study with the code of IR.UMSHA.REC.1401.265. The studies were conducted in accordance with the local legislation and institutional requirements. Written informed consent for participation was not required from the participants or the participants’ legal guardians/next of kin in accordance with the national legislation and institutional requirements.

## Author contributions

AM: Conceptualization, Formal analysis, Writing – original draft, Writing – review & editing. FS: Conceptualization, Writing – original draft, Writing – review & editing. MA: Data curation, Methodology, Writing – original draft, Writing – review & editing. RB: Investigation, Writing – original draft, Writing – review & editing.
